# *TGFBR2* and *BAX* Mononucleotide Tract Mutations, Microsatellite Instability, and Prognosis in 1072 Colorectal Cancers

**DOI:** 10.1371/journal.pone.0025062

**Published:** 2011-09-20

**Authors:** Kaori Shima, Teppei Morikawa, Mai Yamauchi, Aya Kuchiba, Yu Imamura, Xiaoyun Liao, Jeffrey A. Meyerhardt, Charles S. Fuchs, Shuji Ogino

**Affiliations:** 1 Department of Medical Oncology, Dana-Farber Cancer Institute and Harvard Medical School, Boston, Massachusetts, United States of America; 2 Channing Laboratory, Department of Medicine, Brigham and Women's Hospital and Harvard Medical School, Boston, Massachusetts, United States of America; 3 Department of Pathology, Brigham and Women's Hospital and Harvard Medical School, Boston, Massachusetts, United States of America; Université Paris-Diderot, France

## Abstract

**Background:**

Mononucleotide tracts in the coding regions of the *TGFBR2* and *BAX* genes are commonly mutated in microsatellite instability-high (MSI-high) colon cancers. The receptor TGFBR2 plays an important role in the TGFB1 (transforming growth factor-β, TGF-β) signaling pathway, and BAX plays a key role in apoptosis. However, a role of *TGFBR2* or *BAX* mononucleotide mutation in colorectal cancer as a prognostic biomarker remains uncertain.

**Methodology/Principal Findings:**

We utilized a database of 1072 rectal and colon cancers in two prospective cohort studies (the Nurses' Health Study and the Health Professionals Follow-up Study). Cox proportional hazards model was used to compute mortality hazard ratio (HR), adjusted for clinical, pathological and molecular features including the CpG island methylator phenotype (CIMP), LINE-1 methylation, and *KRAS*, *BRAF* and *PIK3CA* mutations. MSI-high was observed in 15% (162/1072) of all colorectal cancers. *TGFBR2* and *BAX* mononucleotide mutations were detected in 74% (117/159) and 30% (48/158) of MSI-high tumors, respectively. In Kaplan-Meier analysis as well as univariate and multivariate Cox regression analyses, compared to microsatellite stable (MSS)/MSI-low cases, MSI-high cases were associated with superior colorectal cancer-specific survival [adjusted HR, 0.34; 95% confidence interval (CI), 0.20–0.57] regardless of *TGFBR2* or *BAX* mutation status. Among MSI-high tumors, *TGFBR2* mononucleotide mutation was associated with CIMP-high independent of other variables [multivariate odds ratio, 3.57; 95% CI, 1.66–7.66; p = 0.0011].

**Conclusions:**

*TGFBR2* or *BAX* mononucleotide mutations are not associated with the patient survival outcome in MSI-high colorectal cancer. Our data do not support those mutations as prognostic biomarkers (beyond MSI) in colorectal carcinoma.

## Introduction

Colorectal cancer represents a group of molecularly heterogeneous diseases with different sets of epigenetic and genetic abnormalities. High degree of microsatellite instability (MSI-high) is caused by deficiency of DNA mismatch repair system, and observed in approximately 15% of colorectal cancers. MSI testing is widely used as screening for patients with Lynch syndrome/hereditary nonpolyposis colorectal cancer (HNPCC) [1], [2], [3]. In addition, MSI is generally accepted as a prognostic marker [4], and likely a predictive marker for resistance to 5-fluorouracil [5]. Since Markowitz et al. [6] discovered mutations in the coding mononucleotide repeats of *TGFBR2* in MSI-high colon cancer cells, similar mutations of coding mononucleotide repeats in many other genes (including *BAX*, *MSH3*, *MSH6*, *IGF2R* and *PTEN*) have been found in MSI-high colorectal cancers [3], [7], [8], [9]. Among those genes, mononucleotide coding repeats of *TGFBR2* (A)_10_ and *BAX* (G)_8_ have frequent frameshift mutations resulting in the production of truncated, inactive form of the proteins [3], [10]. TGFB1 (transforming growth factor-β, TGF-β) and its receptor TGFBR2 constitute a signaling pathway that regulates the transcription of many genes, and functions as a tumor suppressor [11], [12], [13], [14] and an immune response regulator [15]. BAX generally promotes apoptosis and antagonizes the effect of BCL2 [16], [17], [18]. Thus, inactivation of TGFBR2 or BAX may contribute to tumor progression.

Several previous studies have examined the prognostic role of *TGFBR2* or *BAX* mononucleotide mutations in MSI-high colorectal cancers, yielding inconclusive results due to limited statistical power in most studies [19], [20], [21], [22], [23], [24], [25] (**Table 1**). All but one previous study [23] examined the prognostic role of *TGFBR2* or *BAX* mononucleotide mutation in less than 100 MSI-high tumors (the number of MSI-high tumors ranging from 16 to 98) [19], [20], [21], [22], [23], [24], [25]. In addition, none of the previous studies [19], [20], [21], [22], [23], [24], [25] has comprehensively examined potential confounding effect of key molecular biomarkers in colorectal cancer, including the CpG island methylator phenotype (CIMP), and *KRAS*, *BRAF* and *PIK3CA* mutations. Thus, the prognostic role of *TGFBR2* or *BAX* mononucleotide mutation in MSI-high tumors still remains uncertain.

**Table 1:** Studies on prognostic significance of *TGFBR2* or *BAX* mononucleotide mutation in MSI-high colorectal cancer.

Ref.	Authors (year)	No. of hospitals	Sample size for MSI determination	Disease stage	Chemotherapy	MSI-high cases			Mononucleotide mutations in MSI-high colorectal cancer						Other molecular covariates and notes
						N	OS events	CS events	*TGFBR2*			*BAX*			
									Cases number/MSI-high cases	5-year survival *P* value by log-rank test (vs. wild type cases)	Multivariate HR (95% CI) *P* value (vs. wild-type cases)	Cases number/MSI-high cases	5-year survival *P* value by log-rank test (vs. wild type cases)	Multivariate HR (95% CI) *P* value (vs. wild -type cases)	
[19]	Iacopetta et al. (1998)	1	210	Dukes' B and C	-	37	-	-	32/37	OS at the end point* 72% (vs. 60%) *p* = 0.21	-	-	-	-	Proximal colon cancer only
[20]	Ionov et al. (2000)	?	508	-	-	67	-	-	31/36	OS: *p* = 0.55	-	19/36	OS: inferior *p*<0.01	-	
[21]	Watanabe et al. (2001)	many	298	II–III	Adjuvant chemo-therapy	73	-	-	48/73	OS: 74% (vs. 46%) *p* = 0.04	-	22/60	-	-	Multivariate model assessed effect of MSS vs. MSI-high and *TGFBR2* mutation. 18q LOH was also assessed.
[22]	Samowitz et al. (2002)	many	1427	I–IV	-	174	-	-	134/170	OS: 72% (vs. 67%) NS	OS: 1.01 (0.54–1.88) NS	63/160	OS: 67% (vs. 71%) NS	OS: 1.33 (0.80–2.21) NS	Cases from 8 county areas
[23]	Fernández-Peralta et al. (2005)	1	155	Dukes' A–D	-	16	-	-	13/16	OS: superior *p* = 0.04	-	6/16	OS: superior *p*<0.001	-	
[24]	Jung et al. (2006)	4	172	II	-	48	11	-	35/45	-	OS: NS (univariate HR only)	29/47	-	-	
[25]	Kim et al. (2007)	many	542	Dukes' B and C	Adjuvant chemo-therapy†	98	26	-	54/98	-	OS (stratified by stage and treatment): 1.26 (0.57–2.80) NS	-	-	-	National Surgical Adjuvant Breast and Bowel Project (NSABP)
	Shima et al. (current study)	many	1072	I–IV	-	162	59	20	117/159	CS: 86% (vs. 90%), *p* = 0.55OS: 64% (vs. 60%), *p* = 0.66	CS: 1.18 (0.29–4.89), *p* = 0.82OS: 0.61 (0.32–1.15), *p* = 0.12	48/158	CS: 92% (vs. 85%), *p* = 0.29OS: 56% (vs. 65%), *p* = 0.39	CS: 0.73 (0.22–2.41), *p* = 0.60OS: 1.46 (0.80–2.65), *p* = 0.22	Tumor molecular covariates include CIMP, LINE-1 methylation, and mutations in *KRAS*, *BRAF* and *PIK3CA*.

CI, confidence interval; CIMP, CpG island methylator phenotype; CS, colorectal cancer-specific survival; HR, hazard ratio; LOH, loss of heterozygosity; MSI, microsatellite instability; MSS, microsatellite stable; NS, not significant; OS, overall survival;

*Including MSS/MSI-low cases.

† The authors (Kim et al. [25]) compared predictive effect of MSI for chemotherapy between the cases registered surgery alone and the cases registered chemotherapy.

We conducted this study to test the hypothesis that *TGFBR2* or *BAX* mononucleotide mutations in colorectal cancer were associated with altered tumor behavior (beyond MSI), utilizing a database of 1072 stage I to IV colorectal cancers in two prospective cohort studies. Our current study represents the first study which utilized a database of prospective cohort studies to test the stated hypothesis. This fact increases generalizability of our study findings. Moreover, because we concurrently assessed clinical, pathologic and tumor molecular variables such as the CpG island methylator phenotype (CIMP), LINE-1 methylation, *KRAS*, *BRAF* and *PIK3CA* mutations, we could evaluate the effect of *TGFBR2* or *BAX* mutation independent of these potential confounders.

## Methods

### Study group

We utilized the database of two prospective cohort studies, the Nurses' Health Study (N = 121,701 women followed since 1976) and the Health Professionals Follow-up Study (N = 51,529 men followed since 1986) [26], [27]. Participants have been sent biennial questionnaires to update information on potential risk factors and to identify newly diagnosed cancers in themselves and their first degree relatives. We collected paraffin-embedded tumor tissue blocks of incident colorectal cancers from hospitals throughout the U.S. where participants with colorectal cancer underwent tumor resection [26], [27]. Clinical characteristics of the cases are described in **Table 2** (on the left, under the column heading “All cases”). There was no significant difference in demographic features between cases with tissue available and those without available tissue among our cohort studies [26]. A majority of cases have previously been characterized for statuses of *TGFBR2*, MSI, CIMP, *KRAS*, *BRAF*, *PIK3CA* and LINE-1 methylation [28], [29], [30], [31]. However, none of our previous studies have analyzed the prognostic significance of mononucleotide mutation of *TGFBR2* or *BAX*. *BAX* mutation has not been analyzed in any of our previous studies. Thus, this study represents a new study utilizing a resource of the existing materials and database, analogous to novel studies using well-described cell lines (e.g., SW480 cell line) or mouse models (e.g., *Apc* min mouse model).

**Table 2:** MSI status and *TGFBR2* or *BAX* mononucleotide tract mutation in colorectal cancer.

Clinical, pathologic or molecular feature	Total N	MSS/MSI-low	MSI-high	*TGFBR2* mononucleotide mutation in MSI-high tumors			*BAX* mononucleotide mutation in MSI-high tumors		
				(−)	(+)	P value	(−)	(+)	P value
All cases	1072	910	162	42	117		110	48	
Sex						0.45			0.51
Female (NHS)	603 (56%)	495 (54%)	108 (67%)	26 (62%)	80 (68%)		72 (65%)	34 (71%)	
Male (HPFS)	469 (44%)	415 (46%)	54 (33%)	16 (38%)	37 (32%)		38 (35%)	14 (29%)	
Mean age ± SD	67.5±8.5	67.2±8.6	69.5±7.3	68.3±7.6	69.7±7.0	0.26	68.7±7.4	70.6±6.4	0.13
Body mass index						0.34			0.62
<30 kg/m^2^	871 (81%)	740 (81%)	131 (81%)	32 (76%)	97 (83%)		88 (80%)	40 (83%)	
≥30 kg/m^2^	200 (19%)	169 (19%)	31 (19%)	10 (24%)	20 (17%)		22 (20%)	8 (17%)	
Family history of colorectal cancer						0.68			0.56
Absent	866 (81%)	743 (82%)	123 (76%)	31 (74%)	90 (77%)		85 (77%)	35 (73%)	
Present	206 (19%)	167 (18%)	39 (24%)	11 (26%)	27 (23%)		25 (23%)	13 (27%)	
Year of diagnosis						0.28			0.68
Prior to 1995	390 (36%)	347 (38%)	43 (26%)	14 (33%)	29 (25%)		31 (28%)	12 (25%)	
1995 to 2004	682 (64%)	563 (62%)	119 (73%)	28 (67%)	88 (75%)		79 (72%)	36 (75%)	
Tumor location						0.12			0.51
Proximal colon (cecum to transverse)	492 (47%)	351 (39%)	141 (87%)	35 (83%)	105 (90%)		95 (86%)	44 (92%)	
Distal colon	337 (32%)	321 (36%)	16 (9.9%)	4 (10%)	11 (9.4%)		11 (10%)	4 (8.3%)	
Rectum	228 (22%)	223 (25%)	5 (3.1%)	3 (7.1%)	1 (0.9%)		4 (3.6%)	0	
Disease stage						0.045			0.014
I	256 (24%)	224 (25%)	32 (20%)	15 (36%)	17 (15%)		26 (24%)	6 (13%)	
II	308 (29%)	221 (24%)	87 (54%)	18 (43%)	66 (56%)		48 (44%)	35 (73%)	
III	282(26%)	256 (28%)	26 (16%)	4 (9.5%)	22 (19%)		21 (19%)	5 (10%)	
IV	146 (14%)	136 (15%)	10 (6.2%)	3 (7.1%)	7 (6.0%)		8 (7.3%)	2 (4.2%)	
unknown	80 (7.5%)	73 (8.0%)	7 (4.3%)	2 (4.8%)	5 (4.3%)		7 (6.4%)	0	
Tumor grade						0.86			0.19
Low	962 (90%)	851 (94%)	111 (69%)	29 (69%)	79 (68%)		78 (71%)	29 (60%)	
High	104 (10%)	53 (5.9%)	51 (31%)	13 (31%)	38 (32%)		32 (29%)	19 (40%)	
CIMP status						0.0010			0.52
CIMP-0	462 (44%)	450 (51%)	12 (7.5%)	3 (7.3%)	9 (7.8%)		9 (8.3%)	3 (6.3%)	
CIMP-low	411 (39%)	382 (43%)	29 (18%)	15 (37%)	12 (10%)		21 (19%)	6 (13%)	
CIMP-high	172 (16%)	53 (6.0%)	119 (74%)	23 (56%)	95 (82%)		78 (72%)	39 (81%)	
*KRAS* mutation						0.016			0.81
(−)	684 (64%)	546 (60%)	138 (86%)	31 (76%)	105 (91%)		93 (86%)	42 (88%)	
(+)	383 (36%)	361 (40%)	22 (14%)	10 (24%)	11 (9.5%)		15 (14%)	6 (13%)	
*BRAF* mutation						0.0049			0.59
(−)	915 (86%)	835 (92%)	80 (50%)	28 (68%)	50 (43%)		55 (50%)	22 (46%)	
(+)	150 (14%)	69 (7.6%)	81 (50%)	13 (32%)	67 (57%)		54 (49%)	26 (54%)	
*PIK3CA* mutation						0.53			0.079
(−)	789 (81%)	679 (82%)	110 (76%)	26 (72%)	82 (77%)		71 (72%)	37 (86%)	
(+)	189 (19%)	154 (18%)	35 (24%)	10 (28%)	24 (23%)		27 (28%)	6 (14%)	
Mean LINE-1 methylation (%) ± SD	62.0±9.4	61.2±9.3	66.1±8.5	66.2±9.8	65.9±8.0	0.83	66.0±8.6	66.0±8.4	0.96

(%) indicates the proportion of cases with a specific clinical, pathologic or molecular feature among all cases, MSS/MSI-Low, *TGFBR2* mutated or *BAX* mutated cases. CIMP, CpG island methylator phenotype; HPFS, Health Professionals Follow-up Study; MSI, microsatellite instability; MSS, microsatellite stable, NHS, Nurses' Health Study; SD, standard deviation.

**Figure 1:**
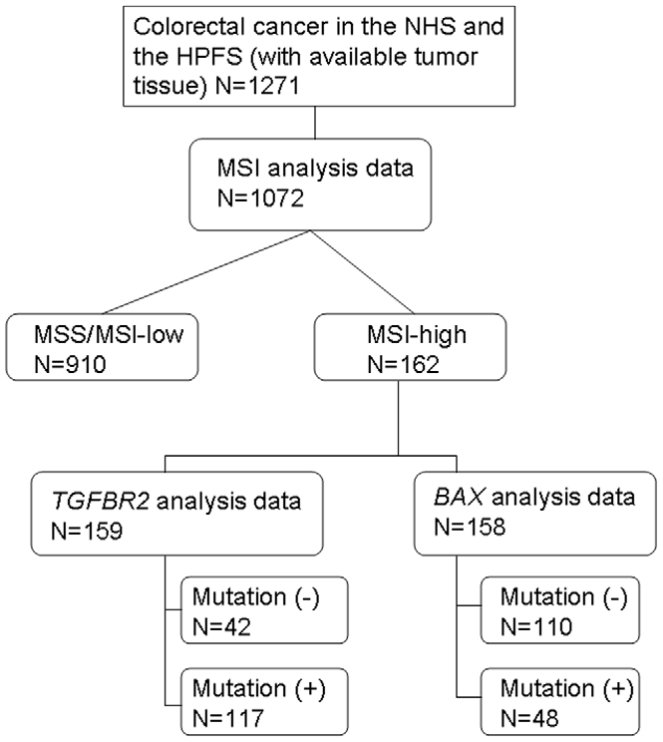
Flow diagram of the current study. Based on the availability of adequate follow-up and tumor molecular data among incident colorectal cancers identified in the Nurses' Health Study (NHS; N = 121,701) and the Health Professionals Follow-up Study (HPFS; N = 51,529), a total of 1072 stage I–IV colorectal cancer cases diagnosed up to 2004 were included. MSI, microsatellite instability; MSS, microsatellite stable.

Hematoxylin and eosin stained tissue sections from all colorectal cancer cases were reviewed by a pathologist (S.O.) unaware of other data. Tumor differentiation was categorized as well-moderate vs. poor (>50% vs. ≤50% glandular areas). We excluded cases which were preoperatively treated. Based on the availability of adequate follow-up and tumor tissue data, 1072 stage I–IV colorectal cancer cases diagnosed up to 2004 were included in the current study (**Figure 1**). Patients were observed until death or June 30 2009, whichever came first. Death of a participant was confirmed by the National Death Index. Returning questionnaire indicated informed consent from all study subjects. Informed consent was obtained from all study subjects. Tissue collection and analyses were approved by the Human Subjects Committees at Harvard School of Public Health and Brigham and Women's Hospital.

### Microsatellite instability (MSI) analysis and detection of *TGFBR2* and *BAX* mononucleotide tract mutations

DNA was extracted from paraffin embedded tissue. MSI analysis was performed using 10 microsatellite markers (D2S123, D5S346, D17S250, BAT25, BAT26, BAT40, D18S55, D18S56, D18S67 and D18S487) [30]. MSI-high was defined as the presence of instability in ≥30% of the markers, and MSI-low/microsatellite stable (MSS) as instability in 0–29% of markers [30]. Mononucleotide tract mutations of *TGFBR2* and *BAX* were examined in MSI-high tumors. Primers and PCR conditions for *TGFBR2* were previously described [28]. Primer sequences for *BAX* were; 5′-(FAM) ATCCAGGATCGAGCAGGGCG-3′ and 5′-ACTCGCTCAGCTTCTTGGTG-3′. PCR condition was preheat at 95°C for 5 min, 45 cycles (at 94-55-72°C for 30-30-30 sec), and extension at 72°C for 2 min. PCR products were electrophoresed and analyzed by ABI 3730 DNA Analyzer (Applied Biosystems, Foster City, CA).

### Pyrosequencing of *KRAS*, *BRAF* and *PIK3CA*

PCR and Pyrosequencing targeted for *KRAS* (codons 12 and 13) [32], *BRAF* (codon 600) [33]and *PIK3CA* (exons 9 and 20) were performed as previously described [29].

### Methylation analyses for CpG islands and LINE-1

Sodium bisulfite treatment and subsequent real-time PCR (MethyLight [34]) were previously validated [35], and performed to quantify promoter methylation in eight CpG islands (*CACNA1G*, *CDKN2A*, *CRABP1*, *IGF2*, *MLH1*, *NEUROG1*, *RUNX3* and *SOCS1*) [36], [37], [38]. CIMP-high was defined as the presence of ≥6/8 methylated markers, CIMP-low as the presence of 1/8 to 5/8 methylated markers, and CIMP-0 as the absence (0/8) of methylated markers [37], [39]. LINE-1 methylation levels were quantified by PCR-Pyrosequencing [40], [41].

### Statistical analysis

We used SAS program (Version 9.1, SAS Institute, Cary, NC) for all statistical analyses. All p values were two-sided. When we perform multiple hypothesis testing (i.e., analyses of molecular correlates and interactions), a p value for statistical significance was adjusted to p = 0.0038 ( = 0.05/13) by Bonferroni correction. The chi-square test (or Fisher's exact test) was performed for categorical variables. The t test assuming unequal variances was done to compare mean age and mean LINE-1 methylation level. For survival analysis, the Kaplan-Meier method and log-rank test were used. For analyses of colorectal cancer-specific mortality, deaths as a result of causes other than colorectal cancer were censored. To control for confounding, we used multivariate stage-matched (stratified) Cox proportional hazards model to compute hazard ratio (HR) of death. To avoid residual confounding and overfitting, disease stage (I, II, III, IV, unknown) was used as a stratifying variable, utilizing the “strata” option in the SAS “proc phreg” command. The multivariate model initially included age at diagnosis (continuous), sex, year of diagnosis (continuous), body mass index (BMI; <30 vs. ≥30 kg/m^2^), family history of colorectal cancer in any first degree relative (present vs. absent), tumor location (proximal vs. distal), tumor differentiation (well-moderate vs. poor), CIMP (high vs. low/CIMP-0), LINE-1 methylation (continuous), *KRAS*, *BRAF* and *PIK3CA*. A backward elimination method with a threshold of p = 0.20 was used to limit the number of variables in the final model and avoid overfitting. For cases with missing information in any of the categorical variables [BMI (0.1%), tumor location (1.0%), tumor grade (0.6%), CIMP (2.5%), *KRAS* (0.5%), *BRAF* (0.7%) and *PIK3CA* (8.8%)], we included those cases in a majority category of a given covariate to avoid overfitting. We confirmed that excluding cases with missing information in any of the covariates did not substantially alter results (data not shown).

A multivariate logistic regression analysis was performed to examine an independent relationship of each covariate with *TGFBR2* mutation (as an outcome variable). The multivariate model initially included a similar, but not the same set of the covariates as the initial Cox model, considering possible cause-effect relationship with *TGFBR2* mutation. Specifically, disease stage and tumor differentiation were likely consequences (rather than causes) of *TGFBR2* mutation. Thus, those variables were not included in the logistic regression model. A backward elimination with a threshold of p = 0.10 was used to select variables in the final model and avoid overfitting.

## Results

### Mononucleotide mutations of *TGFBR2* and *BAX* in MSI-high colorectal cancers

Among 1072 colorectal cancers in the two prospective cohort studies, MSI-high was observed in 162 (15%) tumors. *TGFBR2* and *BAX* mononucleotide tract mutations were detected in 117 (of 159, 74%) and 48 (of 158, 30%) MSI-high tumors, respectively. Among MSI-high tumors, *TGFBR2* mutation was significantly associated with CIMP-high (p = 0.0010) (**Table 2**).

### Multivariate analysis to assess independent relations with *TGFBR2* mutation

We performed multivariate logistic regression analysis to examine whether *TGFBR2* mutation was independently associated with any clinical, pathologic and other molecular variables. In MSI-high tumors, *TGFBR2* mutation was independently associated with CIMP-high [multivariate odds ratio (OR), 3.57; 95% confidence interval (CI), 1.66–7.66; p = 0.0011].

### Mononucleotide mutations of *TGFBR2* and *BAX* and colorectal cancer prognosis

**Figure 2:**
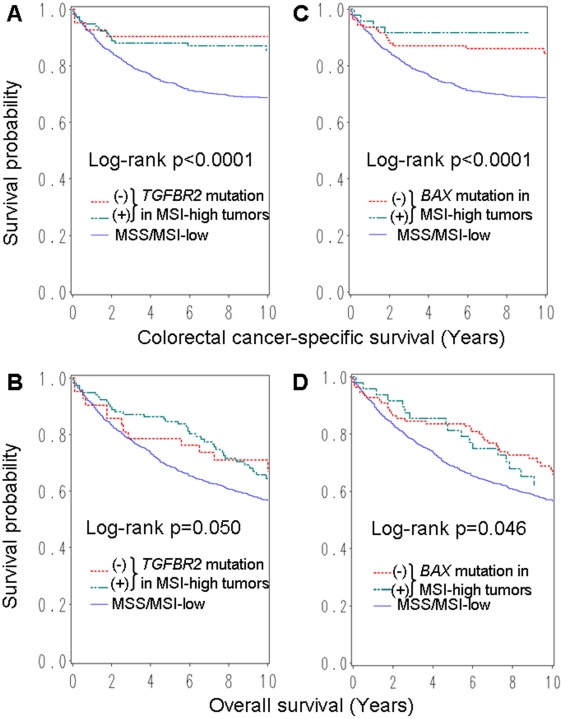
Kaplan-Meier curves according to MSI status and *TGFBR2* or *BAX* mononucleotide mutation in colorectal cancer. Kaplan-Meier curves for colorectal cancer-specific survival (A) and overall survival (B), according to *TGFBR2* mononucleotide mutation status. Regardless of *TGFBR2* status, MSI-high cases were associated with longer survival. Kaplan-Meier curves for colorectal cancer-specific survival (C) and overall survival (D), according to *BAX* mononucleotide mutation status. Regardless of *BAX* status, MSI-high cases were associated with longer survival. MSI, microsatellite instability; MSS, microsatellite stable.

During adequate follow-up (11.6 years of median follow-up of censored cases), there were 505 deaths including 302 colorectal cancer-specific deaths. Among all cases, MSI-high was significantly associated with longer colorectal cancer-specific survival compared to MSS/MSI-low cancers by log-rank test (p<0.0001), univariate and multivariate Cox regression analysis (adjusted HR, 0.34; 95% CI, 0.20–0.57; p<0.0001) (**Table 3**). When we separately examined *TGFBR2*-mutated MSI-high cases and *TGFBR2*-wildtype MSI-high cases, both groups showed significantly longer colorectal cancer-specific survival compared to MSS/MSI-low cases (**Figure 2**, **Table 3**). When we separately examined *BAX*-mutated MSI-high cases and *BAX*-wildtype MSI-high cases, both groups showed significantly longer colorectal cancer-specific survival compared to MSS/MSI-low cases (**Figure 2**, **Table 3**). In overall mortality analyses, although somewhat attenuated, results showed similar trends (**Table 3**). Among MSI-high cases, patient survival did not significantly differ by *TGFBR2* or *BAX* mutation status.

**Table 3:** MSI status, *TGFBR2* and *BAX* mononucleotide tract mutation and survival of colorectal cancer patients.

		Colorectal cancer-specific mortality			Overall mortality		
	Total N	Deaths/person-years	Univariate HR (95% CI)	Multivariate stage-matched HR (95% CI)	Deaths/person-years	Univariate HR (95% CI)	Multivariate stage-matched HR (95% CI)
MSS/MSI-low tumors	910	282/8015	1 (referent)	1 (referent)	445/8015	1 (referent)	1 (referent)
MSI-high tumors	162	20/1468	0.37 (0.24–0.58)	0.34 (0.20–0.57)	60/1468	0.72 (0.55–0.94)	0.64 (0.47–0.89)
MSI-high tumors							
*TGFBR2* mutation (−)	42	4/390	0.30 (0.11–0.79)	0.29 (0.10–0.83)	17/390	0.79 (0.48–1.28)	0.79 (0.47–1.34)
*TGFBR2* mutation (+)	117	16/1060	0.41 (0.25–0.67)	0.35 (0.20–0.62)	42/1060	0.69 (0.50–0.95)	0.59 (0.41–0.86)
*BAX* mutation (−)	110	16/999	0.44 (0.27–0.73)	0.36 (0.20–0.62)	38/999	0.67 (0.48–0.93)	0.57 (0.39–0.84)
*BAX* mutation (+)	48	4/443	0.25 (0.09–0.66)	0.30 (0.11–0.83)	21/443	0.84 (0.54–1.30)	0.82 (0.50–1.32)

The multivariate, stage-matched (stratified) Cox regression model initially included the *TGFBR2* mutation or *BAX* mutation variable, sex, age at diagnosis, year of diagnosis, tumor location, body mass index, family history of colorectal cancer, tumor grade, CIMP, *KRAS*, *BRAF*, *PIK3CA* and LINE-1 methylation. A backward elimination with a threshold of p = 0.20 was used to select variables in the final models. Stage adjustment (I, II, III, IV, unknown) was done using the “strata” option in the SAS “proc phreg” command.

CI, confidence interval; HR, hazard ratio; MSI, microsatellite instability; MSS, microsatellite stable.

We compared colorectal cancer specific and overall survival between *TGFBR2*-mutated MSI-high cases and *TGFBR2*-wildtype MSI-high cases (or between *BAX*-mutated MSI-high cases and *BAX*-wild type MSI-high cases). There was no significant difference between the two groups (**Table 1**).

## Discussion

We conducted this study to examine the prognostic significance of mononucleotide tract mutations in the coding regions of *TGFBR2* or *BAX* in MSI-high colorectal cancers. We utilized two prospective cohort studies with a large number of clinically and molecularly well-annotated colorectal cancer cases with adequate follow-up. Our result showed that MSI-high tumors were associated with indolent tumor behavior regardless of *TGFBR2* or *BAX* mononucleotide mutation status, independent of CIMP and other key tumor molecular biomarkers. Nonetheless, it may be of interest to examine interactions between these molecular alterations and dietary and lifestyle factors if there is a hypothesis in evolving science of molecular pathological epidemiology [42], [43].

It should be noted that small studies are more prone to “publication bias” than large studies [44]. This phenomenon of publication bias occurs because studies with null findings have a higher likelihood of being unwritten and unpublished compared to those with significant results. Compared to small studies (e.g., studies with a sample size of <200 cancers) with null data, large studies with null data are more likely published. As a result, large studies are less prone to publication bias than small studies. Furthermore, academic pressures might force investigators to design small studies which are easy to complete and get data for haste publications, which might contribute to bias [45], [46], [47]. Therefore, we should weigh more on large-scale studies when we evaluate the published literature on prognostic significance of any biomarker such as *TGFBR2* or *BAX* mononucleotide mutation. Publishing null data in well-powered studies [44], [48], [49], [50], [51], [52] are important because publishing significant results in small underpowered studies leads to publication bias.

Our data are generally consistent with some of previous studies [19], [22], [24], [25] (**Table 1**). Watanabe et al. [21] used stage II and III cases that underwent adjuvant chemotherapy, and reported that, *TGFBR2* mutation was associated with improved 5-year overall survival among 73 MSI-high tumors. In another study [20], among 67 MSI-high tumors, *BAX* mutation was associated with poor prognosis. In an underpowered study by Fernández-Peralta et al. [23], among 16 MSI-high tumors, both *TGFBR2* mutation and *BAX* mutation were associated with better prognosis. The largest study (total N = 1427; 170 MSI-high cancers) by Samowitz et al. [22] showed no prognostic role of *TGFBR2* or *BAX* mutations among MSI-high colorectal cancer cases, in agreement with our current study - the second largest study to date and the only study which examined other key tumor molecular biomarkers such as CIMP, LINE-1 methylation and *KRAS*, *BRAF* and *PIK3CA* mutations.

Studying somatic molecular changes and molecular correlates is important in cancer research towards personalized medicine [53], [54], [55], [56]. The CpG island methylator phenotype (CIMP) has been established as an epigenomic molecular classifier of colorectal cancer [57], [58], [59], [60], [61], [62], [63], [64], [65], [66], [67], [68], [69], [70], [71]. In the past, Iacopetta et al. [20] showed no significant association between *KRAS* mutation and *TGFBR*2 mutation. We assessed the association between tumor molecular variables (CIMP, LINE-1, *KRAS*, *BRAF* and *PIK3CA*) and *TGFBR2* mutation and did not find significant relation between *TGFBR2* mutation and *KRAS* or *BRAF* mutation. Interestingly, we have found that, among MSI-high tumors, *TGFBR*2 mutation was associated with CIMP-high, independent of clinical and other molecular features. A recent study [72] has reported that genetic variants in the TGFB1 pathway related genes (*MAPK1*, *RUNX1* and *RUNX2*) are associated with CIMP-high colon cancer. Further studies are needed to elucidate the exact mechanism of the relationship between CIMP and the TGFB1 pathway.

There are limitations in this study. For example, data on cancer treatment were limited. Nonetheless, it is unlikely that chemotherapy use substantially differed according to *TGFBR2* or *BAX* mutation status in tumor, since such data were typically unavailable for treatment decision making. As another limitation, beyond cause of mortality, data on cancer recurrences were unavailable in these cohort studies. Nonetheless, given median follow-up of over 11 years for censored cases, colorectal cancer-specific survival might be a reasonable surrogate of colorectal cancer-specific outcome.

There are advantages in utilizing the database of the two prospective cohort studies, the Nurses' Health Study and the Health Professionals Follow-up Study, to examine prognostic significance of tumor biomarkers. Anthropometric measurements, family history, cancer staging, and other clinical, pathologic, and tumor molecular data were prospectively collected, blinded to patient outcome [26]. Cohort participants who developed cancer were treated at hospitals throughout the U.S., and thus more representative colorectal cancers in the U.S. population than patients in one to a few academic hospitals. There were no demographic difference between cases with tumor tissue analyzed and those without tumor tissue analyzed [26]. Finally, our rich tumor database enabled us to simultaneously assess pathologic and tumor molecular correlates and control for potential confounding by the tumor molecular features.

In conclusion, our large tumor database has shown that, compared to MSS/MSI-low cases, MSI-high colorectal cancer is associated with longer cancer-specific survival, regardless of *TGFBR2* or *BAX* mononucleotide tract mutation status. The importance of large-scale studies cannot be overemphasized because, compared to large studies, small studies are much more prone to publication bias, which can mislead clinical practice.

## References

[1] Boland CR, Goel A (2010). Microsatellite instability in colorectal cancer.. Gastroenterology.

[2] Markowitz SD, Bertagnolli MM (2009). Molecular origins of cancer: Molecular basis of colorectal cancer.. N Engl J Med.

[3] Iacopetta B, Grieu F, Amanuel B (2010). Microsatellite instability in colorectal cancer.. Asia Pac J Clin Oncol.

[4] Popat S, Hubner R, Houlston RS (2005). Systematic review of microsatellite instability and colorectal cancer prognosis.. J Clin Oncol.

[5] de la Chapelle A, Hampel H (2010). Clinical relevance of microsatellite instability in colorectal cancer.. J Clin Oncol.

[6] Markowitz S, Wang J, Myeroff L, Parsons R, Sun L (1995). Inactivation of the type II TGF-beta receptor in colon cancer cells with microsatellite instability.. Science.

[7] Rampino N, Yamamoto H, Ionov Y, Li Y, Sawai H (1997). Somatic frameshift mutations in the BAX gene in colon cancers of the microsatellite mutator phenotype.. Science.

[8] Shin KH, Park YJ, Park JG (2001). PTEN gene mutations in colorectal cancers displaying microsatellite instability.. Cancer Lett.

[9] Duval A, Hamelin R (2002). Mutations at coding repeat sequences in mismatch repair-deficient human cancers: toward a new concept of target genes for instability.. Cancer Res.

[10] Abdel-Rahman WM, Georgiades IB, Curtis LJ, Arends MJ, Wyllie AH (1999). Role of BAX mutations in mismatch repair-deficient colorectal carcinogenesis.. Oncogene.

[11] Biswas S, Chytil A, Washington K, Romero-Gallo J, Gorska AE (2004). Transforming growth factor beta receptor type II inactivation promotes the establishment and progression of colon cancer.. Cancer Res.

[12] Mishra L, Shetty K, Tang Y, Stuart A, Byers SW (2005). The role of TGF-beta and Wnt signaling in gastrointestinal stem cells and cancer.. Oncogene.

[13] Bacman D, Merkel S, Croner R, Papadopoulos T, Brueckl W (2007). TGF-beta receptor 2 downregulation in tumour-associated stroma worsens prognosis and high-grade tumours show more tumour-associated macrophages and lower TGF-beta1 expression in colon carcinoma: a retrospective study.. BMC Cancer.

[14] Chowdhury S, Ammanamanchi S, Howell GM (2009). Epigenetic Targeting of Transforming Growth Factor beta Receptor II and Implications for Cancer Therapy.. Mol Cell Pharmacol.

[15] Ogino S, Galon J, Fuchs CS, Dranoff G (2011). Cancer immunology-analysis of host and tumor factors for personalized medicine.. Nat Rev Clin Oncol.

[16] Grabowski P, Sturm I, Schelwies K, Maaser K, Buhr HJ (2006). Analysis of neuroendocrine differentiation and the p53/BAX pathway in UICC stage III colorectal carcinoma identifies patients with good prognosis.. Int J Colorectal Dis.

[17] Nehls O, Okech T, Hsieh CJ, Enzinger T, Sarbia M (2007). Studies on p53, BAX and Bcl-2 protein expression and microsatellite instability in stage III (UICC) colon cancer treated by adjuvant chemotherapy: major prognostic impact of proapoptotic BAX.. Br J Cancer.

[18] Hector S, Prehn JH (2009). Apoptosis signaling proteins as prognostic biomarkers in colorectal cancer: a review.. Biochim Biophys Acta.

[19] Iacopetta BJ, Welch J, Soong R, House AK, Zhou XP (1998). Mutation of the transforming growth factor-beta type II receptor gene in right-sided colorectal cancer: relationship to clinicopathological features and genetic alterations.. J Pathol.

[20] Ionov Y, Yamamoto H, Krajewski S, Reed JC, Perucho M (2000). Mutational inactivation of the proapoptotic gene BAX confers selective advantage during tumor clonal evolution.. Proc Natl Acad Sci U S A.

[21] Watanabe T, Wu TT, Catalano PJ, Ueki T, Satriano R (2001). Molecular predictors of survival after adjuvant chemotherapy for colon cancer.. N Engl J Med.

[22] Samowitz WS, Curtin K, Neuhausen S, Schaffer D, Slattery ML (2002). Prognostic implications of BAX and TGFBRII mutations in colon cancers with microsatellite instability.. Genes Chromosomes Cancer.

[23] Fernandez-Peralta AM, Nejda N, Oliart S, Medina V, Azcoita MM (2005). Significance of mutations in TGFBR2 and BAX in neoplastic progression and patient outcome in sporadic colorectal tumors with high-frequency microsatellite instability.. Cancer Genet Cytogenet.

[24] Jung B, Smith EJ, Doctolero RT, Gervaz P, Alonso JC (2006). Influence of target gene mutations on survival, stage and histology in sporadic microsatellite unstable colon cancers.. Int J Cancer.

[25] Kim GP, Colangelo LH, Wieand HS, Paik S, Kirsch IR (2007). Prognostic and predictive roles of high-degree microsatellite instability in colon cancer: a National Cancer Institute-National Surgical Adjuvant Breast and Bowel Project Collaborative Study.. J Clin Oncol.

[26] Chan AT, Ogino S, Fuchs CS (2007). Aspirin and the risk of colorectal cancer in relation to the expression of COX-2.. N Engl J Med.

[27] Morikawa T, Kuchiba A, Yamauchi M, Meyerhardt JA, Shima K (2011). Association of CTNNB1 (beta-catenin) alterations, body mass index, and physical activity with survival in patients with colorectal cancer.. JAMA.

[28] Ogino S, Kawasaki T, Ogawa A, Kirkner GJ, Loda M (2007). TGFBR2 mutation is correlated with CpG island methylator phenotype in microsatellite instability-high colorectal cancer.. Hum Pathol.

[29] Nosho K, Kawasaki T, Ohnishi M, Suemoto Y, Kirkner GJ (2008). PIK3CA mutation in colorectal cancer: relationship with genetic and epigenetic alterations.. Neoplasia.

[30] Ogino S, Nosho K, Kirkner GJ, Kawasaki T, Meyerhardt JA (2009). CpG island methylator phenotype, microsatellite instability, BRAF mutation and clinical outcome in colon cancer.. Gut.

[31] Ogino S, Nosho K, Kirkner GJ, Kawasaki T, Chan AT (2008). A cohort study of tumoral LINE-1 hypomethylation and prognosis in colon cancer.. J Natl Cancer Inst.

[32] Ogino S, Kawasaki T, Brahmandam M, Yan L, Cantor M (2005). Sensitive sequencing method for KRAS mutation detection by Pyrosequencing.. J Mol Diagn.

[33] Ogino S, Kawasaki T, Kirkner GJ, Loda M, Fuchs CS (2006). CpG island methylator phenotype-low (CIMP-low) in colorectal cancer: possible associations with male sex and KRAS mutations.. J Mol Diagn.

[34] Eads CA, Danenberg KD, Kawakami K, Saltz LB, Blake C (2000). MethyLight: a high-throughput assay to measure DNA methylation.. Nucleic Acids Res.

[35] Ogino S, kawasaki T, Brahmandam M, Cantor M, Kirkner GJ (2006). Precision and performance characteristics of bisulfite conversion and real-time PCR (MethyLight) for quantitative DNA methylation analysis.. J Mol Diagn.

[36] Ogino S, Cantor M, Kawasaki T, Brahmandam M, Kirkner G (2006). CpG island methylator phenotype (CIMP) of colorectal cancer is best characterised by quantitative DNA methylation analysis and prospective cohort studies.. Gut.

[37] Nosho K, Irahara N, Shima K, Kure S, Kirkner GJ (2008). Comprehensive biostatistical analysis of CpG island methylator phenotype in colorectal cancer using a large population-based sample.. PLoS ONE.

[38] Weisenberger DJ, Siegmund KD, Campan M, Young J, Long TI (2006). CpG island methylator phenotype underlies sporadic microsatellite instability and is tightly associated with BRAF mutation in colorectal cancer.. Nat Genet.

[39] Ogino S, Kawasaki T, Kirkner GJ, Kraft P, Loda M (2007). Evaluation of markers for CpG island methylator phenotype (CIMP) in colorectal cancer by a large population-based sample.. J Mol Diagn.

[40] Ogino S, Kawasaki T, Nosho K, Ohnishi M, Suemoto Y (2008). LINE-1 hypomethylation is inversely associated with microsatellite instability and CpG methylator phenotype in colorectal cancer.. Int J Cancer.

[41] Irahara N, Nosho K, Baba Y, Shima K, Lindeman NI (2010). Precision of Pyrosequencing assay to measure LINE-1 methylation in colon cancer, normal colonic mucosa and peripheral blood cells.. J Mol Diagn.

[42] Ogino S, Stampfer M (2010). Lifestyle factors and microsatellite instability in colorectal cancer: The evolving field of molecular pathological epidemiology.. J Natl Cancer Inst.

[43] Ogino S, Chan AT, Fuchs CS, Giovannucci E (2011). Molecular pathological epidemiology of colorectal neoplasia: an emerging transdisciplinary and interdisciplinary field.. Gut.

[44] Ogino S, Nosho K, Irahara N, Shima K, Baba Y (2009). Prognostic significance and molecular associations of 18q loss of heterozygosity: a cohort study of microsatellite stable colorectal cancers.. J Clin Oncol.

[45] Fanelli D (2010). Do pressures to publish increase scientists' bias? An empirical support from US States Data.. PLoS One.

[46] Ioannidis JP (2005). Why most published research findings are false.. PLoS Med.

[47] Ioannidis JP (2005). Contradicted and initially stronger effects in highly cited clinical research.. JAMA.

[48] Bertagnolli MM, Redston M, Compton CC, Niedzwiecki D, Mayer RJ (2011). Microsatellite Instability and Loss of Heterozygosity at Chromosomal Location 18q: Prospective Evaluation of Biomarkers for Stages II and III Colon Cancer–A Study of CALGB 9581 and 89803.. J Clin Oncol.

[49] Ogino S, Meyerhardt JA, Irahara N, Niedzwiecki D, Hollis D (2009). KRAS mutation in stage III colon cancer and clinical outcome following intergroup trial CALGB 89803.. Clin Cancer Res.

[50] Shima K, Morikawa T, Baba Y, Nosho K, Suzuki M (2011). MGMT promoter methylation, loss of expression and prognosis in 855 colorectal cancers.. Cancer Causes Control.

[51] Shima K, Nosho K, Baba Y, Cantor M, Meyerhardt JA (2011). Prognostic significance of CDKN2A (p16) promoter methylation and loss of expression in 902 colorectal cancers: cohort study and literature review.. Int J Cancer.

[52] Ng K, Ogino S, Meyerhardt JA, Chan JA, Chan AT (2011). Relationship Between Statin Use and Colon Cancer Recurrence and Survival: Results From CALGB 89803.. J Natl Cancer Inst.

[53] Ogino S, Goel A (2008). Molecular classification and correlates in colorectal cancer.. J Mol Diagn.

[54] Franci C, Gallen M, Alameda F, Baro T, Iglesias M (2009). Snail1 protein in the stroma as a new putative prognosis marker for colon tumours.. PLoS One.

[55] Langlois MJ, Bergeron S, Bernatchez G, Boudreau F, Saucier C (2010). The PTEN phosphatase controls intestinal epithelial cell polarity and barrier function: role in colorectal cancer progression.. PLoS One.

[56] Shi T, Mazumdar T, Devecchio J, Duan ZH, Agyeman A (2010). cDNA microarray gene expression profiling of hedgehog signaling pathway inhibition in human colon cancer cells.. PLoS One.

[57] Curtin K, Slattery ML, Samowitz WS (2011). CpG island methylation in colorectal cancer: past, present and future.. Pathology Research International.

[58] Toyota M, Ahuja N, Ohe-Toyota M, Herman JG, Baylin SB (1999). CpG island methylator phenotype in colorectal cancer.. Proc Natl Acad Sci U S A.

[59] Samowitz W, Albertsen H, Herrick J, Levin TR, Sweeney C (2005). Evaluation of a large, population-based sample supports a CpG island methylator phenotype in colon cancer.. Gastroenterology.

[60] Kim JC, Choi JS, Roh SA, Cho DH, Kim TW (2010). Promoter Methylation of Specific Genes is Associated with the Phenotype and Progression of Colorectal Adenocarcinomas.. Ann Surg Oncol.

[61] Kim JH, Shin SH, Kwon HJ, Cho NY, Kang GH (2009). Prognostic implications of CpG island hypermethylator phenotype in colorectal cancers.. Virchow Arch.

[62] Barault L, Charon-Barra C, Jooste V, de la Vega MF, Martin L (2008). Hypermethylator phenotype in sporadic colon cancer: study on a population-based series of 582 cases.. Cancer Res.

[63] Zlobec I, Bihl M, Foerster A, Rufle A, Lugli A (2011). Comprehensive analysis of CpG Island Methylator Phenotype (CIMP)-high, -low, and -negative colorectal cancers based on protein marker expression and molecular features.. J Pathol.

[64] Dahlin AM, Palmqvist R, Henriksson ML, Jacobsson M, Eklof V (2010). The Role of the CpG Island Methylator Phenotype in Colorectal Cancer Prognosis Depends on Microsatellite Instability Screening Status.. Clin Cancer Res.

[65] de Maat MF, Narita N, Benard A, Yoshimura T, Kuo C (2010). Development of sporadic microsatellite instability in colorectal tumors involves hypermethylation at methylated-in-tumor loci in adenoma.. Am J Pathol.

[66] de Vogel S, Wouters KA, Gottschalk RW, van Schooten FJ, de Goeij AF (2011). Dietary methyl donors, methyl metabolizing enzymes, and epigenetic regulators: diet-gene interactions and promoter CpG island hypermethylation in colorectal cancer.. Cancer Causes Control.

[67] Ang PW, Loh M, Liem N, Lim PL, Grieu F (2010). Comprehensive profiling of DNA methylation in colorectal cancer reveals subgroups with distinct clinicopathological and molecular features.. BMC Cancer.

[68] Hughes LA, Simons CC, van den Brandt PA, Goldbohm RA, de Goeij AF (2011). Body size, physical activity and risk of colorectal cancer with or without the CpG island methylator phenotype (CIMP).. PLoS One.

[69] Schernhammer ES, Giovannucci E, Baba Y, Fuchs CS, Ogino S (2011). B vitamins, methionine and alcohol intake and risk of colon cancer in relation to BRAF mutation and CpG island methylator phenotype (CIMP).. PLoS One.

[70] Tanaka N, Huttenhower C, Nosho K, Baba Y, Shima K (2010). Novel Application of Structural Equation Modeling to Correlation Structure Analysis of CpG Island Methylation in Colorectal Cancer.. Am J Pathol.

[71] Teodoridis JM, Hardie C, Brown R (2008). CpG island methylator phenotype (CIMP) in cancer: Causes and implications.. Cancer Lett.

[72] Slattery ML, Lundgreen A, Herrick SJ, Caan BJ, Potter JD (2011). Associations between genetic variation in RUNX1, RUNX2, RUNX3, MAPK1, and eIF4E and risk of colon and rectal cancer: Additional support for a TGF-{beta} signaling pathway.. Carcinogenesis.

